# Towards Reversible High-Voltage Multi-Electron Reactions in Alkali-Ion Batteries Using Vanadium Phosphate Positive Electrode Materials

**DOI:** 10.3390/molecules26051428

**Published:** 2021-03-06

**Authors:** Edouard Boivin, Jean-Noël Chotard, Christian Masquelier, Laurence Croguennec

**Affiliations:** 1 Laboratoire de Réactivité et de Chimie des Solides, CNRS-UMR 7314, Université de Picardie Jules Verne, CEDEX 1, F-80039 Amiens, France; edouard.boivin@univ-lille.fr (E.B.); jean-noel.chotard@u-picardie.fr (J.-N.C.); christian.masquelier@u-picardie.fr (C.M.); 2 CNRS, Université Bordeaux, Bordeaux INP, ICMCB UMR 5026, F-33600 Pessac, France; 3 RS2E, Réseau Français sur le Stockage Electrochimique de l’Energie, FR CNRS 3459, CEDEX 1, F-80039 Amiens, France; 4 ALISTORE-ERI European Research Institute, FR CNRS 3104, CEDEX 1, F-80039 Amiens, France

**Keywords:** batteries, positive electrode, vanadium phosphates, covalent vanadyl bond, mixed anion

## Abstract

Vanadium phosphate positive electrode materials attract great interest in the field of Alkali-ion (Li, Na and K-ion) batteries due to their ability to store several electrons per transition metal. These multi-electron reactions (from V^2+^ to V^5+^) combined with the high voltage of corresponding redox couples (e.g., 4.0 V vs. for V^3+^/V^4+^ in Na_3_V_2_(PO_4_)_2_F_3_) could allow the achievement the 1 kWh/kg milestone at the positive electrode level in Alkali-ion batteries. However, a massive divergence in the voltage reported for the V^3+^/V^4+^ and V^4+^/V^5+^ redox couples as a function of crystal structure is noticed. Moreover, vanadium phosphates that operate at high V^3+^/V^4+^ voltages are usually unable to reversibly exchange several electrons in a narrow enough voltage range. Here, through the review of redox mechanisms and structural evolutions upon electrochemical operation of selected widely studied materials, we identify the crystallographic origin of this trend: the distribution of PO_4_ groups around vanadium octahedra, that allows or prevents the formation of the vanadyl distortion (O^…^V^4+^=O or O^…^V^5+^=O). While the vanadyl entity massively lowers the voltage of the V^3+^/V^4+^ and V^4+^/V^5+^ couples, it considerably improves the reversibility of these redox reactions. Therefore, anionic substitutions, mainly O^2−^ by F^−^, have been identified as a strategy allowing for combining the beneficial effect of the vanadyl distortion on the reversibility with the high voltage of vanadium redox couples in fluorine rich environments.

## 1. Introduction

**Figure 1:**
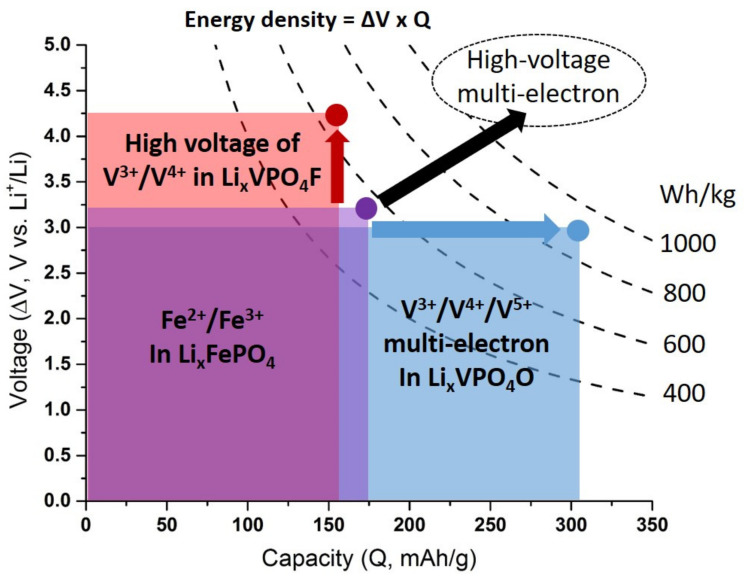
Voltage vs. capacity plot for LiFePO_4_ (**purple**), LiVPO_4_F (**red**), LiVPO_4_O (**blue**). Combining the high voltage of LiVPO_4_F with the high capacity of the multi-electron redox in LiVPO_4_O could allow the achievement of higher energy density through high voltage multi-electron redox. The dash lines represent constant energy densities in Wh/kg of active positive electrode material.

In the 2000s, the research on polyanion compounds as positive electrode materials was mainly motivated by the interesting properties of the low cost triphylite LiFePO_4_ [1,2,3,4,5] (olivine-type structure) providing long-term structural stability, essential for extensive electrochemical cycling and safety issues. In this material, the high voltage for the Fe^2+^/Fe^3+^ redox couple delivered in LiFePO_4_ (i.e., 3.45 V vs. Li^+^/Li vs. ca. 2.2 V in oxides) is due to the inductive effect of the phosphate group. Further exploitation of the inductive effect with fluorine and/or sulfate has led to materials such as LiFeSO_4_F (Tavorite or Triplite structure) delivering an even higher voltage than LiFePO_4_ (i.e., 3.6 V and 3.9 V vs. Li^+^/Li for Tavorite and Triplite structures, respectively) [6,7]. However, these materials suffer from a deficit of capacity compared to the current best commercially available Li-ion positive electrode materials. Li_2_FeSiO_4_ has been proposed to overcome this issue by triggering both Fe^2+^/Fe^3+^ and Fe^3+^/Fe^4+^ redox couples but the strong structural changes involved seem to be detrimental to long-term performances [8]. To the best of our knowledge, this material is the only one providing a multi-electron reaction (i.e., exchange of more than one electron per transition metal) in iron-based polyanion systems while vanadium phosphate materials offer numerous such examples. Indeed, the ability of vanadium to be stabilized in the large range of oxidation states, from V^2+^ to V^5+^ (e.g., from V^3+^ in Li_2_VPO_4_O to V^5+^ in VPO_4_O) [9,10,11,12,13] combined with the rather high voltage of the corresponding redox couples (e.g., 4.25 V vs. Li^+^/Li for V^3+^/V^4+^ in LiVPO_4_F) [14] could allow the achievement of high energy density thanks to reversible high-voltage multi-electron redox in Alkali-ion batteries (Figure 1).

**Figure 2:**
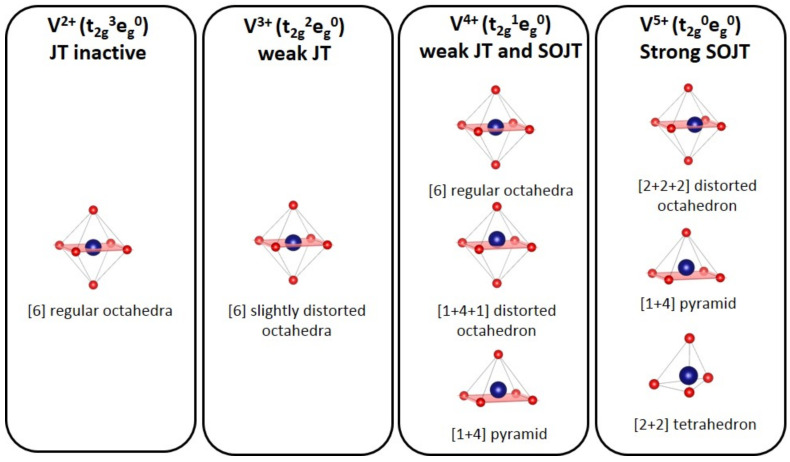
Stable environments of vanadium according to its oxidation state [15]. The number in square brackets correspond to the number of “equivalent bonds”.

However, depending on the geometry of the VO*_n_* polyhedra, the positions of the V^3+^/V^4+^ and V^4+^/V^5+^ redox couples massively change. For instance, Tavorite LiVPO_4_F operates at 4.25 V vs. Li^+^/Li while in the homeotype LiVPO_4_O, the apparent same V^3+^/V^4+^ redox couple is activated at 2.3 V vs. Li^+^/Li. This large difference cannot be attributed only to the inductive effect, Li site energy or even cation-cation repulsion (i.e., main mechanisms reported to govern the voltage of a given redox couple): it is actually due to the vanadyl distortion observed in LiVPO_4_O and not in LiVPO_4_F. The first order Jahn Teller (FOJT) distortion is known to be weak in d^1^ (V^4+^) and d^2^ (V^3+^) and even inexistent for d^3^ (V^2+^) and d^0^ (V^5+^) cations (Figure 2). Therefore, the second order Jahn Teller (SOJT) effect drives the distortion of the V^4+^ and V^5+^ polyhedra while this distortion can be prevented for V^4+^ in a mixed O^2−^/F^−^ environment. This wide range of oxidation states for vanadium cations (V^2+^, V^3+^, V^4+^ and V^5+^) and the extended panel of environments that they can adopt (regular octahedra, distorted octahedra, square pyramids and tetrahedra) with very different electronic configurations depending on the ligand distribution (JT active or JT inactive) confer to the vanadium phosphate a very rich crystal chemistry.

Beyond their fascinating electrochemical properties, vanadium phosphate materials possess very interesting catalytic and magnetic properties. The relation between structures and these properties was already reviewed by Raveau’s group 20 years ago [15] and despite the existence of several reviews on polyanionic structures in Li and Na-ion batteries [16,17,18,19], or even specific to vanadyl phosphates (i.e., A_x_(VO)PO_4_) [20,21], none of them focused on the relation between electrochemical properties and crystallographic structure in vanadium phosphates. Therefore, this article aims at clarifying this relation in order to unveil the structural features that dictate the redox voltage in such compounds. Through the fine description of the redox mechanism and the structural evolution observed during cycling of some widely studied materials (NASICON Na_3_V_2_(PO_4_)_3_, anti-NASICON Li_3_V_2_(PO_4_)_3_, Na_3_V_2_(PO_4_)_2_F_3x_O_x_, and Tavorite-like LiVPO_4_F_1−x_O_x_) we propose to sort all the vanadium phosphates reported as positive electrode materials for Li and Na-ion batteries according to the distribution of phosphate groups around the vanadium polyhedra. This classification gives a holistic picture of such systems and allows for identifying the strategies available to tend towards reversible high-voltage multi-electron reactions in Alkali-ion batteries.

## 2. Irreversible Multi-Electron Reactions in NASICON and Anti-NASICON A_x_V_2_(PO_4_)_3_ (A = Li, Na) Structures

**Figure 3:**
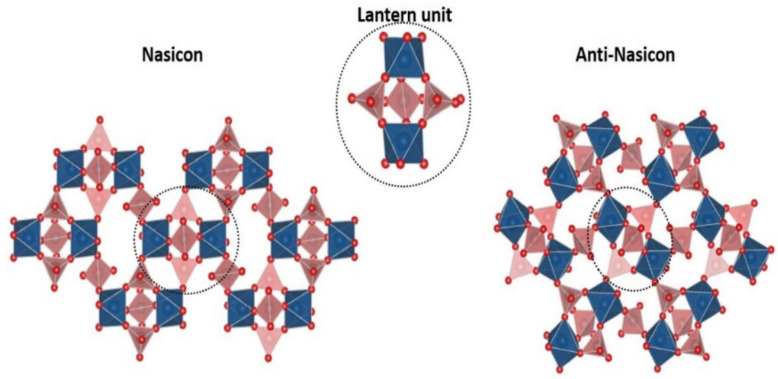
Structural relationship between Nasicon (**left**) and anti-Nasicon (**right**) structures adapted from ref. [18].

The NASICON (Na-super ionic conductor) and anti-NASICON structures have the general formula A_x_M_2_(XO_4_)_3_ (with M = Fe, Ti, Sc, Hf, V, Ti, Zr, etc. or mixtures of them and X = W, P, S, Si, Mo or mixtures of them) [22,23]. These versatile structures have provided a great playground for solid state chemists. Manthiram and Goodenough demonstrated experimentally the inductive effect which modulates the voltage of the Fe^3+^/Fe^2+^ redox couple in NASICON [24] which is at the origin of all advances on polyanion materials as positive electrode materials for Alkali-ion batteries. The crystallographic arrangements of NASICON and anti-NASICON are closely related. Indeed, they are built on a three-dimensional framework of VO_6_ octahedra sharing all their corners with PO_4_ tetrahedra and conversely forming basic V_2_(PO_4_)_3_ repeating units commonly named “lantern units” (Figure 3). The connectivity of the lantern units generates different ion conduction paths, vanadium environments and hence different electrochemical properties.

**Figure 4:**
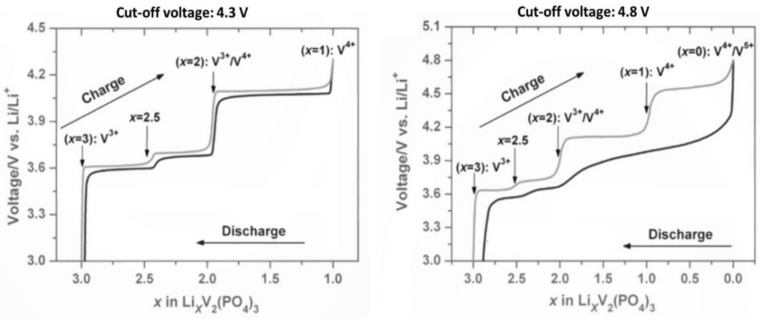
Electrochemical signature of Li_3_V_2_(PO_4_)_3_ cycled between (left) 3.0 and 4.3 V vs. Li^+^/Li or between (right) 3.0 and 4.8 V vs. Li^+^/Li adapted from ref. [29]. Reproduced with permission from Rui et al., Journal of Power Sources; published by Elsevier, 2014.

In the structure of the anti-NASICON polymorph of Li_3_V_2_(PO_4_)_3_, the lithium ions fully occupy three crystallographic sites (one tetrahedral Li(1)O_4_ and two pseudo tetrahedral Li(2)O_4_O and Li(3)O_4_O sites) [25,26,27]. The electrochemical extraction of lithium from Li_3_V_2_(PO_4_)_3_ occurs according to several biphasic reactions involving the V^3+^/V^4+^ redox couple at 3.6, 3.7 and 4.1 V vs. Li^+^/Li and then the V^4+^/V^5+^ one at 4.5 V vs. Li^+^/Li (Figure 4).

**Figure 5:**
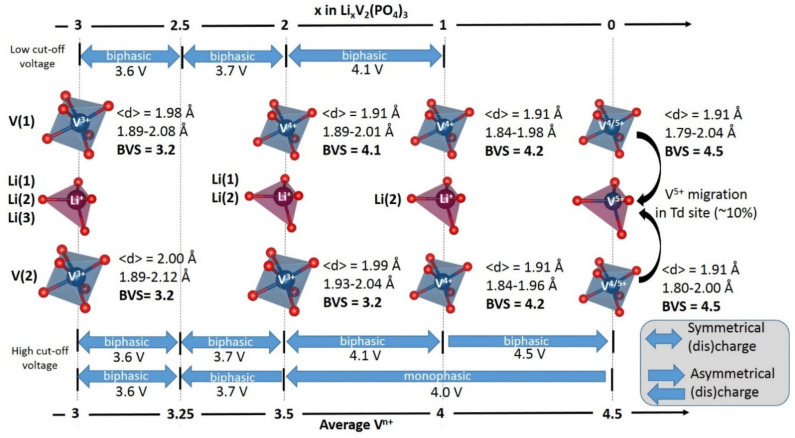
Structural evolution during Lithium extraction/insertion from/into Li_3_V_2_(PO_4_)_3_ [27,32].

Nazar and coworkers [27] studied the complex phase diagram involved during lithium extraction from Li_3_V_2_(PO_4_)_3_ through X-ray and neutron diffraction (XRD and ND) and solid state nuclear magnetic resonance spectroscopy (NMR), as summarized in Figure 5. The first delithiation step leads to the formation of Li_2_._5_V_2_(PO_4_)_3_ with partial depopulation of the pseudo tetrahedral (Li(3)O_4_O) site and to a complex short range ordering of V^3+^/V^4+^ cations [28]. The following delithiation stage affects only the remaining Li(3) ions to yield to Li_2_V_2_(PO_4_)_3_ characterized by lithium/vacancies and V^3+^/V^4+^ orderings as suggested by diffraction. The further oxidation of vanadium allows reaching the V^4+^-rich LiV_2_(PO_4_)_3_ phase in which only one Li site remains (Li(2)) as fully occupied. In this phase, there are two very similar crystallographic sites for vanadium ((V(1)-O = V(2)-O = 1.91 Å in average).

The last process leading to the V_2_(PO_4_)_3_ composition is kinetically more limited with a large over-potential (around 500 mV) [27]. At this state of charge, the environments of vanadium (with a mixed valence V^4+^/V^5+^) become more distorted, although without significant modification compared to the average V-O distances observed in the V^IV^-rich LiV_2_(PO_4_)_3_ phase. This extraction/insertion process is asymmetrical as the lithium ordering observed for LiV_2_(PO_4_)_3_ during charge is not observed during discharge. A disordered Lithium re-intercalation is observed until the Li_2_V_2_(PO_4_)_3_ composition is reached [30]. This asymmetrical mechanism is not observed for a lower cut-off voltage (i.e., 4.3 V when the V^4+^/V^5+^ redox couple is not activated, see Figure 4). Under this electrochemical cycling conditions the charge and discharge superimposes [31]. That was tentatively explained, in ref. [27], by the occurrence of Lithium/vacancies ordering observed in LiV_2_(PO_4_)_3_ which involves an ordered depopulation of Lithium, whereas from the disordered fully delithiated phase the lithium is free to be inserted randomly until the Li_2_V_2_(PO_4_)_3_ composition is recovered. More recently, operando XAS at V K-edge investigation of this irreversible mechanism suggested the formation of anti-site Li/V defects at high voltage (Figure 5) providing V^5+^ with a much more stable tetrahedral environment than its initial distorted octahedral one [32].

In Li_3_V_2_(PO_4_)_3_, the electronically insulating phosphate groups isolate the valence electrons of transition metals within the lattices resulting in low intrinsic electronic conductivities—a trend common to all polyanion compounds. Therefore, the use of carbon coating or/and doping elements are required to improve the electrochemical performances: all the works applying these strategies are reviewed in ref [29]. The majority of these studies reports good performances only in the small voltage range (i.e., 3.0–4.3 V vs. Li^+^/Li, in which only the V^3+^/V^4+^ is activated). Indeed, due to the strong distortion of vanadium environments and the Li/V anti-site defects generated, the kinetic limitations of the V^4+^/V^5+^ process is difficult to overcome.

The lithium insertion into Li_3_V_2_(PO_4_)_3_ reveals a complex series of reactions as well, to reach Li_5_V_2_(PO_4_)_3_ by activating the V^2+^/V^3+^ redox couple [33]. The whole lithium insertion process into Li_3+x_V_2_(PO_4_)_3_ involves four consecutive two-phase regions to reach Li_5_V_2_(PO_4_)_3_. Approximately 0.5 Li^+^ is inserted at every potential plateau around 1.95, 1.86, 1.74 and 1.66 V vs. Li^+^/Li [26]. To the best of our knowledge, the crystallographic details of this complex mechanism have never been fully studied yet.

The anti-NASICON polymorph of Li_3_V_2_(PO_4_)_3_ is most thermodynamically stable but Gaubicher et al. [34] obtained the NASICON form by Na^+^/Li^+^ ionic exchange from Na_3_V_2_(PO_4_)_3_. This material reveals a similar electrochemical signature compared to the one of Na_3_V_2_(PO_4_)_3_ with a single plateau until the LiV_2_(PO_4_)_3_ composition at 3.7 V vs. Li^+^/Li. The crystal structure of Na_3_V_2_(PO_4_)_3_ was originally reported by Delmas et al. [35] 40 years ago using the standard rhombohedral unit cell, S.G. *R-3c*. Since then, Na_3_V_2_(PO_4_)_3_ has almost always been reported to adopt the rhombohedral symmetry with a partial occupancy of both Na(1) (6*b* Wyckoff position) and Na(2) (18*e* Wyckoff position) sodium sites. However, a recent article reveals that a *C2/c* space group is more appropriate to describe this structure at room temperature and below due to Sodium-vacancies ordering [36] within five sites (one 4*a* and four others 8*f*) fully occupied. Several transitions between 10 and 230 °C involving four distinct phases (α ordered, β and β′ with incommensurate modulations and γ disordered) were also reported. The transition between the α and β forms occurring close to the ambient temperature (i.e., 27 °C) may impact the sodium diffusion and a fortiori the electrochemical performances while the vanadium environment is hardly impacted by this phase transition. In both cases the VO_6_ entities are slightly distorted with distances ranging between 1.97 and 2.03 Å for the rhombohedral description (2.00 Å in average on a single vanadium site) or 1.94 and 2.06 Å for the monoclinic one (2.00 Å in average on the three vanadium sites).

**Figure 6:**
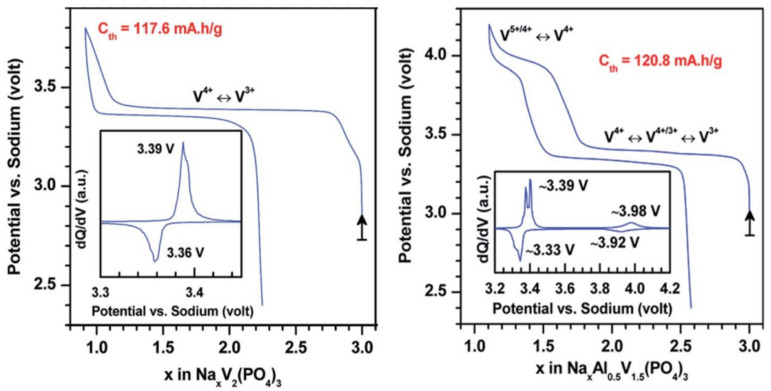
High voltage signature of Nasicon Na_3_V_2_(PO_4_)_3_ and Na_3_V_1.5_Al_0.5_(PO_4_)_3_, adapted from ref. [39]. Reproduced from Ref. [39] with permission from the Centre National de la Recherche Scientifique (CNRS) and The Royal Society of Chemistry.

The electrochemical sodium extraction from Na_3_V_2_(PO_4_)_3_ occurs at a 3.4 V vs. Na^+^/Na according to a biphasic reaction until the NaV_2_(PO_4_)_3_ composition is reached (Figure 6). The structure of this V^4+^ phase, reported by Jian et al. [37], keeps a NASICON framework (Rhombohedral, *R*$\bar{3}$*c*) with only one fully occupied sodium site (6*b* Wyckoff site). During the sodium extraction, the V-O distances in VO_6_ octahedra undergo an inequivalent shortening leading to distorted VO_6_ octahedra (with 3 V-O distances at 1.86 Å and three others at 1.95 Å). The electrochemical extraction of the third sodium has never been reported despite the apparent successful chemical extraction realized by Gopalakrishnan et al. [38]. However, they did not report the detailed structure of this mixed-valence V_2_(PO_4_)_3_.

Even though the third Na^+^ of Na_3_V_2_(PO_4_)_3_ doesn’t seem electrochemically removable, the V^4+^/V^5+^ redox couple in the NASICON was reported to lie at around 4 V vs. Na^+^/Na thanks to the partial substitution of a part of Vanadium by Aluminum [39], Iron [40] or Chromium [41]. The Aluminum substituted material presents two advantages as it allows an increase in the capacity due to the lower weight of aluminum compared to vanadium (and also iron and chromium) as well as to reach the mixed valence V^4+^/V^5+^ state at rather high voltage (i.e., 4.0 V vs. Na^+^/Na, see Figure 6). However, in the Al^3+^ substituted compound, the V^4+^/V^5+^ capacity is limited contrarily to that observed in Na_3_VCr(PO_4_)_3_ where nearly 1.5 electrons/vanadium are exchanged [42]. At room temperature, this redox process induces a rapid degradation of the performance due to the migration of vanadium into the vacant Na site, while at lower temperature (i.e., −15 °C), vanadium is pinned in its original position leading to a rather reversible process is observed [43]. The V^4+^/V^5+^ redox couple has also been reported in Na-rich NASICON such as Na_4_MnV(PO_4_)_3_ [44,45,46,47]. From this compound, ca. 3 Na^+^ are exchanged based on the V^3+^/V^4+^, Mn^2+^/Mn^3+^ and then V^4+^/V^5+^ redox achieving a capacity of 155 mAh/g. However, this latter appears to be poorly reversible inducing a higher irreversible capacity upon discharge during which the highly polarized S-shape voltage profile contrasts with staircase charge curve [45,46,47].

The replacement of a (PO_4_)^3−^ group in Na_3_V_2_(PO_4_)_3_ by 3 F^−^ leads to the Na_3_V_2_(PO_4_)_2_F_3_ composition, often named as a “NASICON composition” but its crystal structure is fundamentally different.

## 3. Irreversible Multi-Electron Reactions in Na_3_V_2_(PO_4_)_2_F_3_

The first physico-chemical investigation of the Na_3_M_2_(PO_4_)_2_F_3_ system was conducted 20 years ago by Le Meins et al. [48]. They demonstrated a great compositional tunability of this framework which can accommodate many trivalent cations in octahedral sites (M = Al, V, Cr, Fe and Ga) and proposed the description of the structure of the vanadium phase in the P4_2_/mnm space group. Later, a combined synchrotron X-ray and neutron diffraction investigation revealed a tiny orthorhombic distortion at room temperature [49].

**Figure 7:**
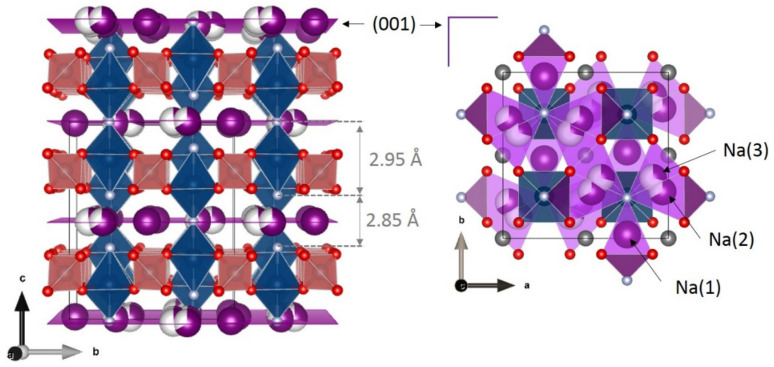
Structure of Na_3_V_2_(PO_4_)_2_F_3_ (**left**) and sodium distribution (**right**) [49].

The *Amam* space group (i.e., *S.G.* #63, *Cmcm*) used leads to a different sodium distribution in the cell with three Na sites, one *4c* fully occupied and two *8f* partially occupied (approximatively distributed as 1/3:2/3) (Figure 7). The host structure is composed of V_2_O_8_F_3_ bi-octahedra sharing a fluorine aligned along the [001] direction and connected to each other through PO_4_ tetrahedra aligned in parallel with the (001) plane (Figure 7). The VO_4_F_2_ octahedra in this structure are non-centrosymmetric and hence vanadium does not occupy the inversion center. Indeed, a displacement along the c direction leads to two slightly different V-F bonds (V-F(1) = 1.968(6) Å and V-F(2) = 1.981(2) Å).

**Figure 8:**
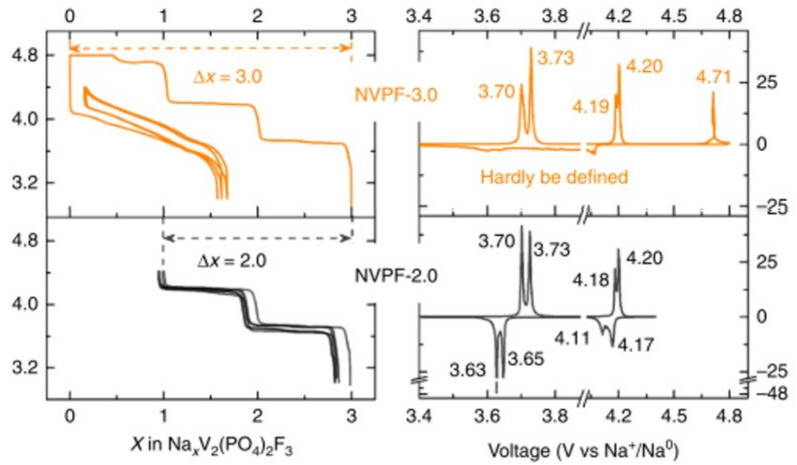
Galvanostatic electrochemical voltage-composition data of Na_3_V_2_(PO_4_)_2_F_3_ at C/10 per exchanged ion and the corresponding derivative curve in the 3.0–4.4 V or 3.0–4.8 V voltage range adapted from ref. [51]. Reproduced with permission from Guochan Yan et al., Nature Communications; published by Springer Nature, 2019.

Slow electrochemical galvanostatic cycling shows the presence of four distinct reversible voltage-composition features at 3.70, 3.73, 4.18 and 4.20 V vs. Na^+^/Na (Figure 8) suggesting a complex phase diagram upon sodium extraction/reinsertion [50].

**Figure 9:**
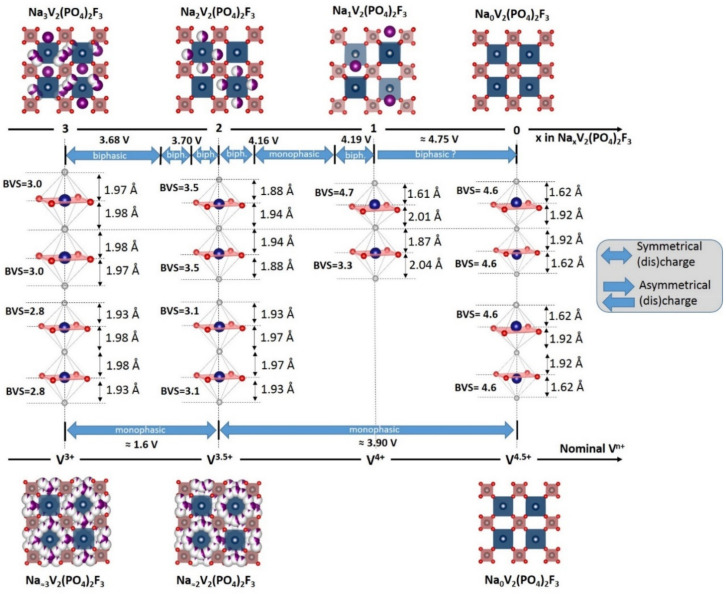
Evolution of vanadium environments and Na/vacancies ordering upon cycling of Na_3_V_2_(PO_4_)_2_F_3_ [51,52,54].

The operando synchrotron XRD investigation conducted by Bianchini et al. [52] is summarized in Figure 9. The phase diagram involves several intermediate phases of compositions Na_x_V_2_(PO_4_)_2_F_3_ with x = 2.4, 2.2, 2, 1.8 and 1.3 before the NaV_2_(PO_4_)_2_F_3_ is reached. During extraction of the first sodium, an alternation between ordered and disordered phases (Na^+^/vacancy and/or V^3+^/V^4+^ ordering and disordering) is observed. The superstructure peaks observed for the Na_2.4_V_2_(PO_4_)_2_F_3_ disappear for Na_2.2_V_2_(PO_4_)_2_F_3_ and the diffraction pattern of Na_2_V_2_(PO_4_)_2_F_3_ reveals the reappearance of a series of additional contributions non-indexed in the tetragonal cell. In the V^3+^-rich phase, the two symmetrically inequivalent V-F bonds are very similar and as the oxidation of vanadium is increased, two kinds of bonds gradually appear as a short one at 1.88 Å and a longer one at 1.94 Å, whereas the equatorial V-O bonds decrease uniformly (from 1.99 to 1.95 Å). The extraction of the second sodium also involves intermediate phases at x = 1.8 and x = 1.3 accompanied by the disappearance of the superstructure peaks and finally leads to the formation of NaV_2_(PO_4_)_2_F_3_. This phase contains a single Na site and two vanadium sites conferring to vanadium cations two very different environments despite an average oxidation state of V^4+^. Indeed, the BVS calculation suggests the formation of a V^3+^-V^5+^ pair in bi-octahedra at this composition (Figure 9). The investigation of the redox mechanism involved during sodium extraction was conducted by Broux et al. [53] through operando XANES at V K-edge. They evidenced that V^4+^ starts to disproportionate from Na_2_V_2_(PO_4_)_2_F_3_ and hence the formation of V^3+^-V^5+^ pairs are confirmed for Na_1_V_2_(PO_4_)_2_F_3_.

Kang and coworkers predicted that the extraction of the third Na^+^ towards the mixed valence V^4+^/V^5+^ V_2_(PO_4_)_2_F_3_ composition would occur only at very high voltage (ca. 4.9 V vs. Na^+^/Na) [55]. This was confirmed experimentally by Tarascon’s group, under sever oxidative conditions (i.e., potentiostatic step at 4.8 V vs. Na^+^/Na see Figure 8) in an optimized electrolyte [51]. In this structure, vanadium is displaced from the inversion center of the VO_4_F_2_ octahedra in such a way as to generate a short 1.62 Å and a longer 1.92 Å V-F bond lengths within the bi-octahedra. However, these extreme cycling conditions imply an irreversible reaction and only 2 Na^+^ could be reinserted upon discharge down to 3.0 V, according to a solid solution mechanism, the third Na^+^ being reinserted at much lower voltage (i.e., 1.6 V vs. 3.7 V for the same composition range upon charge). The subsequent charge/discharge allows for the reversible extraction/insertion of 3 Na^+^ in a wide voltage range (1.0–4.4 V vs. Na^+^/Na). This new β-Na_3_V_2_(PO_4_)_2_F_3_ polymorph exhibits a different symmetry, different V-X bond lengths and a disordered Na distribution (see Figure 9) which bears strong resemblance with the one of the high temperature phase (T > 400 K) [49,54]. Due to the low voltage associated to the reinsertion of third Na^+^, only 2 Na^+^ can be exchanged in a real battery system where the third one acts as an alkali reservoir to compensate for the solid electrolyte interface (SEI) formation at negative electrode [51], which allows offering up to 460 Wh/kg in full cell vs. hard carbon (+18% compared with a conventional α-Na_3_V_2_(PO_4_)_2_F_3_), corresponding to the highest energy density reported so far in Na-ion battery [56].

Most of the Na_3_V_2_(PO_4_)_2_F_3_ materials reported as stoichiometric in the literature actually present various amounts of vanadyl-type defects (i.e., partial substitution of F^−^ by O^2−^ with a charge compensation by partial oxidation of V^3+^-F into vanadyl V^4+^=O) impacting on the electrochemical performance. Several authors studied in detail the crystallographic changes generated by this substitution in Na_3_V_2_(PO_4_)_2_F_3−x_O_x_ (with 0 ≤ x ≤ 0.5 [54], 0 ≤ x ≤ 2 [55] and x = 1.6 [57,58]). This oxidation has strong effects on the local environments of vanadium and on the sodium distribution and appears to be beneficial for enhancing the charge rates of the battery. Kang’s group [57] was the first to investigate the performance of Na_3_V_2_(PO_4_)_2_F_1.4_O_1.6_ (i.e., V^3.8+^) as a positive electrode material and reported high charge and discharge rate capabilities, assigned to a low activation energy for Na^+^ diffusion (~350 meV) inside the framework, despite the poor electronic conductivity (~2.4 × 10^−12^ S.cm^−1^) and its great cycling stability was assigned to the small volume change during sodium extraction/insertion (~3%). The same group later published a promising result about the computed voltage for the extraction of the third sodium around 4.7 V for Na_3_V_2_(PO_4_)_2_F_1.5_O_1.5_ [55] (lower than the one computed up to 4.9 V in Na_3_V_2_(PO_4_)_2_F_3_) and experimentally realized the reversible exchange of more than one electron per vanadium at high voltage (ca. 3.8V vs. Na^+^/Na in average) with a symmetrical charge/discharge profile and an improved capacity retention.

## 4. Low Voltage Multi-Electron Reactions in Tavorites Li_x_VPO_4_Y (Y = O, F and/or OH)

Tavorite-type compositions of general formula A_x_MXO_4_Y are a third class of very interesting polyanion structures in which A is an alkali cation (i.e., Li, Na and 0 ≤ x ≤ 2) and M a metal (i.e., Mg, Al, Ti, V, Fe, Mn, Zn or mixture of them). The polyanionic group, XO_4_, is either PO_4_ or SO_4_ and the bridging anion, Y, is a halide, hydroxide, oxygen, H_2_O group or a mixture of them [18]. The multiple redox center combined with this double inductive effect bring a strong interest at both practical and fundamental levels as it allows scanning a wide range of working voltages, from 1.5 V for Ti^3+^/Ti^4+^ in LiTiPO_4_O [59] to 4.26 V for the V^3+^/V^4+^ redox couple in LiVPO_4_F [60]. The high voltages provided by the V^3+^/V^4+^ and V^4+^/V^5+^ redox couples confer high theoretical energy densities to the vanadium-based Tavorite compositions.

**Figure 10:**
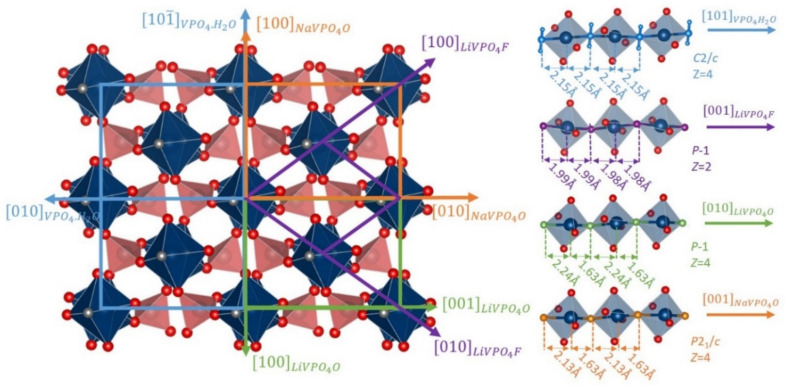
Structural relationships between different Tavorite-type materials.

The crystal structure of Tavorite-like materials can be described in either triclinic (*P-1*, with Z = 2 or Z = 4) or monoclinic (*C2/c* or *P2_1_/c*) systems [14,61,62]. Tavorite-like therefore gathers Tavorite (*P-1*, Z = 2, LiVPO_4_F and LiVPO_4_OH), Montebrasite (*P-1*, Z = 4, LiVPO_4_O), Maxwellite (*C2/c*, NaVPO_4_F and HVPO_4_.OH) and even Talisite (*P2_1_/c*, NaVPO_4_O) structures. Their crystallographic arrangements present common features which can be broadly described as vanadium octahedra (VO_4_Y_2_) sharing a bridging anion Y in order to form infinite chains $\left[-Y-{\mathrm{VO}}_{4}-Y-\right]{\;}_{\infty }$. These chains are connected to each other through PO_4_ tetrahedra sharing their four oxygen atoms with four vanadium octahedra belonging to three different chains. This 3D framework accommodates Li^+^ or Na^+^ in hexagonal channels. The symmetry of vanadium octahedra is dictated by the nature of the V^n+^-Y bond. Indeed, in V^3+^-rich LiVPO_4_F, NaVPO_4_F and VPO_4_·H_2_O, the vanadium is located on an inversion center of the VO_4_Y_2_ octahedra, whereas in V^4+^-rich NaVPO_4_O and LiVPO_4_O a loss of the centrosymmetry of the vanadium environment is observed. Indeed, in the Tavorite-like structure, for an oxidation state of vanadium superior to +3, vanadium likely forms the vanadyl bond resulting from the Jahn Teller activity of V^4+^ (d^1^ t2g^1^eg^0^). This strongly covalent V=O bond can be formed only with oxygen atoms which are not already involved in a covalent PO_4_ group. Only the bridging oxygen, Y, fulfils these requirements and, therefore, in the V^4+^ compounds, an ordering between short and long bonds takes place along the chains (Figure 10). This ordering generates a change of space group (from C2/c for NaVPO_4_F to *P2_1_/c* for NaVPO_4_O) or a doubling of the cell size (Z = 2 for LiVPO_4_F to *Z* = 4 for LiVPO_4_O).

**Figure 11:**
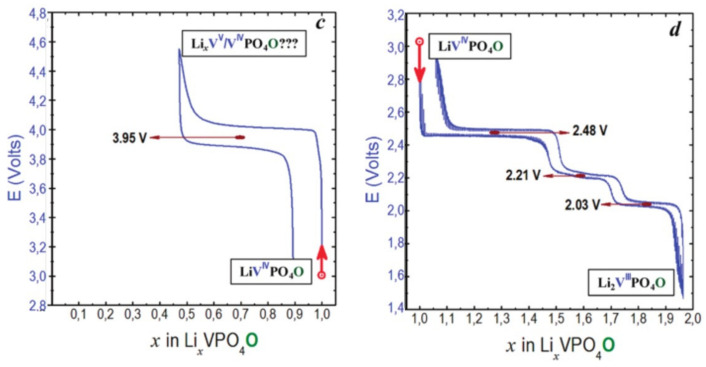
Voltage profile of Li_x_VPO_4_O cycled between 3.0–4.6 V vs. Li^+^/Li (**left**) and between 3.0 and 1.5 V vs. Li^+^/Li in GITT mode (**right**) adapted from ref. [14]. Reproduced with permission from Ateba Mba et al., Chemistry of Materials; published by American Chemical Society, 2012.

The lithium content in Li_x_VPO_4_O can vary from 0 to 2, leading to a capacity of 300 mAh/g at an average voltage of ca. 3.1 V allowing the achievement a stable energy density > 900 Wh/kg using surface engineering and nanosizing strategies [9,10,11,12,13]. However, the large difference between the voltage for oxidation of V^4+^ into V^5+^ (i.e., 3.95 V vs. Li^+^/Li) and that for the reduction in V^4+^ to V^3+^ (around 2.3 V vs. Li^+^/Li) makes this multi-electron reaction unsuitable for a real battery system (Figure 11).

In the high voltage region (i.e., 3.0–4.6 V vs. Li^+^/Li involving the V^4+^/V^5+^ redox couple), the oxidation process occurs via a biphasic mechanism between LiVPO_4_O and VPO_4_O [14]. The crystal structure of this V^5+^ phase (ε-VPO_4_O) is described in a *Cc* space group allowing the formation of vanadyl bonds appearing as shorter than the ones observed in LiVPO_4_O (i.e., 1.59 vs. 1.67 Å, Figure 10). Conversely, the antagonist V^5+…^O bond along the chains elongates from 2.2 Å in LiVPO_4_O to 2.5 Å in VPO_4_O leading to an unconventional increase in the cell volume during lithium extraction (ΔV/V = 4.1%) [63]. This VPO_4_O polymorph can also be obtained while deintercalating the homeotype LiVPO_4_OH (and also VPO_4_·H_2_O), according to an original mechanism [64,65]. Indeed, VPO_4_OH appears instable vs. LiVPO_4_OH and VPO_4_O as this V^4+^-rich phase is not formed upon Li^+^ deintercalation from LiVPO_4_OH. On the contrary, VPO_4_O is formed showing that the V^3+^-O/V^5+^=O redox couple is activated at a constant equilibrium voltage of 3.95 V vs. Li^+^/Li [65]. Indeed, in the VPO_4_OH hypothetical phase the competition between the two highly covalent bonds, V^4+^=O on one side and O-H bond on the other side, would destabilize the V^IV^-O-H sequence, leading to the concomitant extraction of Li^+^ and H^+^ and to the atypical two-electron V^3+^/V^5+^=O redox reaction at a constant voltage. Unfortunately, on the contrary to the two-electron reaction observed in Li_x_VPO_4_O over 3.2 V, which is reversible but not practical, this one observed at a constant high voltage leads to an irreversible phase transformation.

The Li^+^ insertion within LiVPO_4_O involves two intermediate phases, Li_1.5_VPO_4_O and Li_1.75_VPO_4_O, before reaching the Li_2_VPO_4_O [66]. Although this V^3+^-rich composition is described in a triclinic (*P-1*, *Z* = 4) system allowing the formation of a vanadyl-type distortion along the chains, the refined V-O distances do not reveal significant differences between them [63], in agreement with the weak Jahn Teller activity of V^3+^ (d^2^ t2g^2^eg^0^). Lin et al. [67] studied in detail the structural evolutions at the local scale during the lithium insertion in Li_1+x_VPO_4_O, and V K-edge EXAFS shows the disappearance of vanadyl bond for Li_1.5_VPO_4_O and the persistence of the longer antagonist until Li_1.75_VPO_4_O in good agreement with the phase transitions observed (Figure 11).

**Figure 12:**
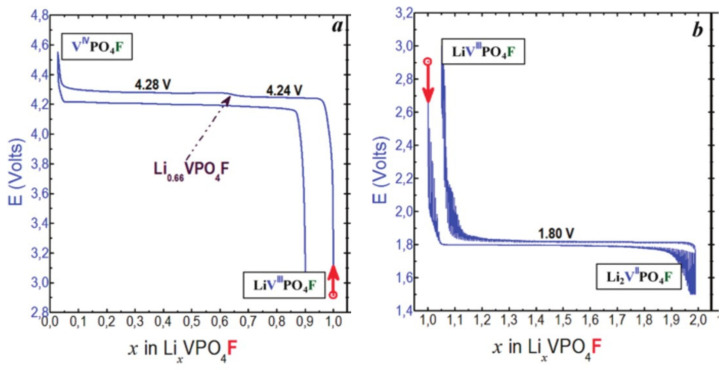
Voltage profile of Li_x_VPO_4_F cycled between 3.0–4.6 V vs. Li^+^/Li (**left**) and between 3.0 and 1.5 V vs Li^+^/Li in GITT mode (**right**) adapted from ref. [14]. Reproduced with permission from Ateba Mba et al., Chemistry of Materials; published by American Chemical Society, 2012.

The investigation of LiVPO_4_F started in 2003 with a series of studies conducted by Barker and co-workers [60,68,69] who highlighted the promising performance of this material. Indeed, the high voltage delivered for the Lithium extraction (4.25 V vs. Li^+^/Li for the V^3+^/V^4+^ redox voltage, Figure 12) and a capacity very close to the theoretical one even at high C-rate confer to this material a higher practical energy density compared to the ones of commercially available LiFePO_4_ and LiCoO_2_ (655 vs. 585 and 525 Wh/kg respectively).

**Figure 13:**
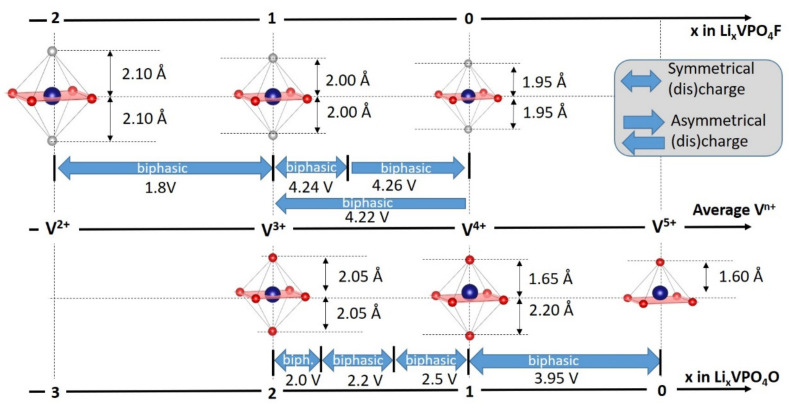
Structural evolution during Lithium extraction/insertion from/into LiVPO_4_F [72] and LiVPO_4_O [63,67].

The Lithium extraction from LiVPO_4_F involves an intermediate phase, Li_2/3_VPO_4_F, and then VPO_4_F through two biphasic reactions. The crystal structure of VPO_4_F was reported by Ellis et al. [70], its *C2/c* space group involving centrosymmetric vanadium octahedra with V-F distances of 1.95 Å (Figure 13) whereas the actual nature of the Li_2/3_VPO_4_F phase is still unclear, although superstructure peaks have been identified and indexed by doubling the b parameter [71]. This intermediate phase is not formed during discharge where a biphasic reaction between the end-member compositions VPO_4_F and LiVPO_4_F takes place [72]. This asymmetric charge/discharge mechanism is not understood at the moment even though it was first attributed by Ellis et al. to the presence of two lithium sites partially occupied (0.8/0.2) in the starting LiVPO_4_F. Nevertheless, this hypothesis was ruled out later by Ateba Mba et al. [67] who localized Lithium in a single fully occupied site. Piao et al. [73] conducted operando V-K edge XANES in order to probe the redox mechanism during delithiation of LiVPO_4_F. By a principal component analysis, three components were required to fit the series of spectra recorded upon charge. This might suggest at least a V^3+^/V^4+^ ordering for Li_2/3_VPO_4_F. The lithium insertion into LiVPO_4_F occurs at low voltage, typical for the V^3+^/V^2+^ redox couple (i.e., 1.8 V vs. Li^+^/Li) through a biphasic reaction leading to the formation of Li_2_VPO_4_F (Figure 12). The structure of Li_2_VPO_4_F is described in a *C2/c* space group with V^2+^ sitting in a centrosymmetric VO_4_F_2_ octahedra with V-F distances at 2.10 Å and equatorial V-O ones at 2.13 Å in average [70] (Figure 13) while Li^+^ ions are distributed between two 8f Wyckoff sites half occupied in LiO_3_F_2_ environments.

Various chemical routes to obtain polycrystalline powders of Tavorite LiVPO_4_F were reported: sol-gel-assisted carbo thermal reduction (CTR) [74], ionothermal [75]. The majority of these reports highlight the difficulty to obtain pure powders (i.e., without anti-NASICON Li_3_V_2_(PO_4_)_3_ impurity) or vanadyl-free compounds. Indeed, a series of recent papers demonstrated, by ^7^Li NMR (and its 2D analogue) and DFT calculations, the presence of various amounts of vanadyl-type defects in crystallographically pure “LiVPO_4_F” [76,77]. Recently, B. Kang and co-workers [78] reported on an ingenious strategy to avoid the fluorine loss during synthesis, using PTFE as an additional fluorine source. The material thus obtained reveals high electrochemical performance with a stable discharge capacity of 120 mAh/g at 10C over 500 cycles. The same group also published for the first time the electrochemical properties of the mixed valence V^3+^/V^4+^ LiVPO_4_F_0.25_O_0.75_ [79]. This strategy aimed at decreasing the difference in voltage between Li insertion and extraction reactions, conferring to the material a high energy density (i.e., 820 Wh/kg) in a reduced voltage range (i.e., 2.0–4.5 V vs. Li^+^/Li) with the activation of the V^3+^/V^4+^ and V^4+^/V^5+^ redox couples, respectively. Further investigation of the LiVPO_4_F-LiVPO_4_O tie-line has allowed several compositions to stabilize in which the competition between ionicity of the V^3+^-F bond and covalency of the V^4+^=O bond distorts the structure, freezes the framework upon Li extraction and hence allows for improved rate capabilities compared with the end-member phases [80,81]. Interestingly, upon Li deintercalation from these materials, the V^4+^=O/V^5+^=O redox couple is triggered first before the V^3+^/V^4+^ is activated in fluorine rich environments leading to the formation of a mixed valence V^3+^-V^5+^ phase at half charge [81]. Although surprising, this redox mechanism is in full agreement with the operating voltage of the end-member phases, the V^4+^/V^5+^ redox couple being activated at 3.95 V in LiVPO_4_O and the V^3+^/V^4+^ redox couple at 4.25 V in LiVPO_4_F due to the absence of vanadyl distortion in LiVPO_4_F and VPO_4_F.

Most of the vanadium phosphates discussed above operate at a rather high V^3+^/V^4+^ redox voltage, suggesting a massive improvement of the energy density delivered while triggering the V^4+^/V^5+^ redox couple. However, materials that operate at such a high V^3+^/V^4+^ voltage are usually unable to reversibly exchange several electrons in a narrow enough voltage range. In the following section we will clarify the crystallographic origin of this trend and identify the strategies able to overcome this issue.

## 5. Towards Reversible High-Voltage Multi-Electron Reactions

Many other vanadium phosphates (as well as pyrophosphates and phosphites, see Table 1) have been stabilized and studied as positive electrode materials for Li(Na)-ion batteries. This article does not aim at providing an exhaustive review of all of them, however careful descriptions of selected systems, provided above, now allow us to generalize and predict part of their properties (especially working voltages, redox mechanisms and structural evolutions) from the only consideration of their crystal structures in their pristine state.

Table 1 highlights the significant divergence in the V^3+^/V^4+^ redox voltages which cannot be attributed to the inductive effect, the cation-cation repulsions or even Li site energy, which are the main reported features impacting the voltage in polyanions [103,104]. Indeed, the voltage for the V^3+^/V^4+^ redox couple in the Tavorite system Li_x_VPO_4_Y (with Y = O or F) varies from 2.4 V for Li_1+x_VPO_4_O (0 ≤ x ≤ 1) to 4.26 V in Li_1-x_VPO_4_F (0 ≤ x ≤ 1). This is attributed to the effect of the highly covalent vanadyl bond which is observed for oxidation states of vanadium strictly superior to 3 in Li_1-x_VPO_4_O, Na_4_VO(PO_4_)_2_ … These structures present a common crystallographic feature: at least one oxygen around vanadium is not involved in a covalent P-O bond and hence could be engaged in a vanadyl bond. In the compounds where the VO_6_ octahedra share all their oxygen atoms with PO_4_ (or P_2_O_7_) groups, the structure of the de-alkalinated V^4+^ phases are vanadyl free with VO_6_ octahedra slightly distorted. The corresponding vanadyl free V^3+^/V^4+^ redox couple is located at 3.9 V in monoclinic Li_3-x_V_2_(PO_4_)_3_ and 4.2 V in Li_1-x_VP_2_O_7_, a much higher voltage than the V^3+^/V^4+^ couple involved in Li_1+x_VPO_4_O polymorphs (around 2.3 V vs. Li^+^/Li).

**Figure 14:**
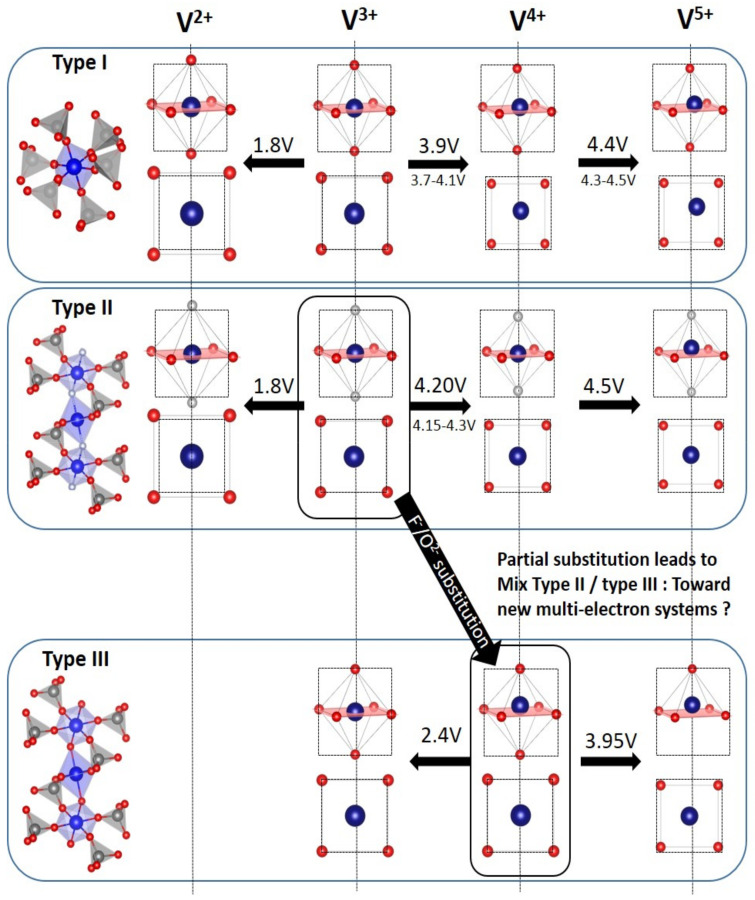
Typical evolution of vanadium environments according to the oxidation state of vanadium for type I, type II and type III materials.

Boudin et al. [15] proposed a classification of the vanadium phosphates into three groups according to size of the “clusters” of vanadium polyhedra ([VO_x_]*_n_* with 1 < *n* < ∞). Although this classification is pertinent to the discussion of the catalytic or magnetic properties of vanadium phosphates, it does not really make sense for a discussion of electrochemical properties. Therefore, we chose to sort these materials considering vanadyl-forbidden (type I and type II) and vanadyl-allowed (type III) structures (summarized in Table 1 and Figure 14):

- For type I materials (e.g., Li_3_V_2_(PO_4_)_3_), in which the vanadyl bond cannot appear due to the involvement of each oxygen atom of VO_6_ octahedra in a PO_4_-type entity, the typical evolution of the vanadium environment upon oxidation (from V^2+^ to V^5+^) follows a quasi-homogeneous shortening of V-O bonds from V^2+^ to V^4+^ and a strong increase in VO_6_ distortion to reach the V^5+^ state with corresponding voltages of 1.8 V vs. Li^+^/Li for V^2+^/V^3+^, 3.9 V vs. Li^+^/Li for V^3+^/V^4+^ and 4.4 V vs. Li^+^/Li for V^4+^/V^5+^ redox couples (on average for all the type I materials reported in Table 1).
- In type II materials (e.g., LiVPO_4_F), at least one of the ligands around vanadium is unshared with a phosphate group and hence would be available to form the vanadyl bond. However, in that case, the nature of this ligand (F^−^ instead of O^2−^) inhibits its formation. From V^2+^ to V^4+^, the evolution of the vanadium environment follows a similar trend with slightly higher voltages than for type I due to the higher ionicity of V-F versus V-O. For V^5+^, for instance in deintercalated Na_3_V_2_(PO_4_)_2_F_3_, a “vanadyl-like” distortion appears with V-F bond length of 1.6 Å and 1.9 Å. Such an F^…^V-F sequence has never been reported elsewhere and the precise nature of the V-F bonds formed is still to be clarified.
- Type III group (e.g., LiVPO_4_O) gathers the structures having at least one oxygen belonging to VO_6_ octahedra available to form the covalent vanadyl bond for vanadium oxidation states higher than 3. In this class of materials, the V^3+^ environments are quasi undistorted. As the oxidation state of vanadium is increased, vanadium leaves the inversion center of the VO_6_ octahedra in order to form the vanadyl bond. The formation of a distorted V^IV^O_6_ octahedra (with typical distances ranging between 1.6 and 2.4 Å along dz^2^ and quasi equivalent equatorial distances around 2 Å) and V^V^O_5_ pyramids (in which the short V=O bond is about 1.6 Å and a shortening of the equatorial distances is observed around 1.8–1.9 Å) are observed. The corresponding voltages appear completely different to those of type I and type II materials: 2.4 V vs. Li^+^/Li for the V^3+^-O/V^4+^=O and 3.95 V vs. Li^+^/Li for the V^4+^=O/V^5+^=O redox couples.

Note that type II materials are crystallographically pseudo type III ones in which the oxygen involved in the vanadyl bond is replaced by Fluorine. Therefore, partial substitution of this fluorine by oxygen leads to mixed type II/III materials—which is actually the case for most of the type III materials, difficult to obtain as vanadyl-free. Extended oxy-fluorine solid solutions were investigated for Na_3_V_2_(PO_4_)_2_F_3−y_O_y_ [54,55,57,58] and for LiVPO_4_F_1-y_O_y_ [79,80,81,105,106]. The particularity of these compounds resides in the redox paradox of vanadium where the V^4+^=O/V^5+^=O is activated at lower voltage than the V^3+^-F/V^4+^-F [55,81,107]. Depending on the distribution of ligands around V, it behaves as type II (V^3+^O_4_F_2_), type III (V^4+^O_4_O_2_) or mixed type II/III (V^3/4+^O_4_OF) [81]. For this latter environment, the V^5+^=O vanadyl-like distortion is allowed upon cycling, promoting the reversibility of the process, but is observed at higher voltage than type III materials thanks to the antagonist fluorine. This highlights the importance of the heteroleptic units formed in statistically distributed or in peculiar O/F ordered compounds which are somewhat difficult to obtain due to the different nature of the V^4+^=O and V^3+^-F bonds promoting their clusterization [108].

This classification makes further sense regarding the ability of each type of vanadium phosphates to reversibly exchange several electrons per transition metal at high voltage and in a narrow enough voltage range. In type III materials multi-electron redox through V^3+^-O/V^4+^=O and V^4+^=O/V^5+^=O couples have often been reported [9,10,11,12]. The oxygen atoms unshared with PO_4_ facilitate the formation of the vanadyl bond allowing for two rather reversible electron processes and thus allow the achievement of cycling stability with energy density higher than 900 Wh/kg [9]. However, this multi-electron reaction cannot be used in a real battery system due to the large voltage difference between the V^3+^-O/V^4+^=O and V^4+^=O/V^5+^=O (ca. 2.5 V) redox couples. Substituting oxygen by fluorine in such a way to obtain LiVPO_4_F_0.75_O_0.25_ allows raising the voltage of the V^3+^/V^4+^ redox and hence reversibly intercalating 1.6 electrons per vanadium in a reduced voltage range [79]. However, this material suffers from rapid capacity fading under such conditions. Since then, the possibility to stabilize multiple compositions along the LiVPO_4_F-LiVPO_4_O tie-line has been demonstrated and a systemic investigation of substitution ratio (i.e., y) vs. the voltage range could allow fixing this issue by controlling the Δx in Li_1±x_VPO_4_F_1−y_O_y_.

In type I and type II materials, while the low voltage V^2+^/V^3+^ (≈1.8 V vs. Li^+^/Li) and high voltage V^3+^/V^4+^ (3.9–4.2 V vs. Li^+^/Li) redox are easily triggered, the V^4+^/V^5+^ redox is rarely reported (see Table 1). Moreover, this latter is often kinetically limited and/or irreversible, most probably due to the structural rearrangements required to provide to V^5+^ cations a satisfying environment. Indeed, the V^5+^ cations are stable either in a pyramidal ([1+4] coordination) or in a tetrahedral ([2+2] coordination) or even in a very distorted octahedral ([2+2+2] coordination) [15] environments. In each case, at least one covalent vanadyl bond must be formed, but this formation is not privileged by the crystallographic arrangements adopted by type I and II materials. In order to provide to the V^5+^ cations with a more stable environment than this distorted octahedral one, migration of V^5+^ in tetrahedral site has been proposed [32]. Therefore, kinetic limitations and/or an irreversible capacity, which can be compensated only at very low voltage (as seen in Na_3_V_2_(PO_4_)_2_F_3_, Li_3_V_2_(PO_4_)_3_, Li_5_V(PO_4_)_2_F_2_ and Li_9_V_3_(P_2_O_7_)_3_(PO_4_)_2_), appear. Although V^5+^ migration in tetrahedral sites has been reported only in Li_3_V_2_(PO_4_)_3_ so far, analyzing the electrochemical response upon subsequent discharge for other compounds gives insight about the nature of the irreversible reaction taking place. For instance, in Na_3_V_2_(PO_4_)_2_F_3_, the Na re-insertion into V_2_(PO_4_)F_3_ (i.e., V^4.5+^) occurs at 3.9 V vs. Na^+^/Na in average, until the composition Na_2_V_2_(PO_4_)_2_F_3_ (V^4.5+^ to V^3.5+^): this voltage range is associated to the Na_3_(V^3+^)-Na_1_(V^4+^) composition range during the previous charge. The further Na insertion occurs at 1.6 V vs. Na^+^/Na until the composition Na_3_V_2_(PO_4_)_2_F_3_ is recovered. Moreover, the length of this low voltage plateau is proportional to the amount of vanadium oxidized above V^4+^ during the previous charge. Therefore, this low voltage feature is more likely to correspond to the reduction in V^3+^ (i.e., ≈1.5 V vs. Na^+^/Na for V^3+^/V^2+^ redox in type I and type II materials) rather than to the reduction in the V^4+^ into V^3+^ (i.e., 3.6–3.9 V vs. Na^+^/Na). This behavior could agree with the presence of V^5+^ in Td sites. Indeed, as seen in transition metal vanadates used as anode in alkali-ion batteries [109], V^5+^_Td_ is not reduced above 1.5 V vs. Li^+^/Li without migrating back in an octahedral site. Therefore, the V^3+^ reduction would occur at a higher voltage than the V^5+^_Td_ reduction. The presence of oxygen in the fluorine site would help in accommodating V^5+^ cation in distorted octahedral site in the charged state. Indeed, it has been shown by theoretical calculations that the partial substitution of fluorine by oxygen in such a way to obtain Na_3_V_2_(PO_4_)_2_F_3−x_O_x_ composition tends to decrease the voltage of extraction of the third Na^+^ cations (from 4.9 V to 4.7 V vs. Na^+^/Na from pure fluoride to oxy-fluoride) leading to the reversible exchange of more than one electron per vanadium with an excellent rate capability [55].

Finally, this review reveals that the versatility of the vanadium chemistry with a large number of stable oxidation states stabilized in very different environments opens the road towards the formation of new materials whose strains imposed by the crystal field give attractive electrochemical properties. While, in the battery field, the search for new polyanion positive electrode materials slows down for few years, maintaining the efforts towards the stabilization of new phases is crucial. LiVPO_4_F_1–x_O_x_ and Na_3_V_2_(PO_4_)_2_F_3–x_O_x_ are the only vanadium phosphate oxy-fluorides studied as positive electrode materials and have shown very promising properties. Further playing with anionic substitution, not only with vanadium phosphate oxy-fluorides but also oxy-nitrides (as recently reported with Na_3_V_2_(PO_3_)_3_N [91]) and even oxy-sulfides, would offer new degrees of flexibility for such versatile polyanion systems and could allow the achievement of high energy density (ca. 1 kWh/kg of active positive electrode material corresponding to ca. 400 Wh/kg at the cell level) through reversible high-voltage multi-electron redox.

## 6. Conclusions

This review has identified the vanadyl distortion as the main feature governing the operating voltage in vanadium phosphates and their ability to reversibly store several electrons per transition metal. The classification of such materials in three groups, according to the nature of the ligands in the vanadium octahedra and to the distribution of PO_4_ around them, has allowed to unveil the strategies to increase their energy density. Indeed, anionic substitutions have led to vanadium phosphate oxy-fluorides which allow to combine the beneficial effect of the vanadyl distortion on the reversibility with the high voltage of vanadium redox couples in fluorine rich environments. Further investigation of these anionic substitutions could allow to tend towards reversible high-voltage multi-electron reactions in Alkali-ion batteries.

## Figures and Tables

**Table 1:** List of the vanadium phosphate, pyro-phosphate and phosphite materials with their redox voltage and corresponding practical capacity based on vanadium redox. More details about the classification of these materials (Type I, II or III) are provided in the text and at the Figure 14.

As Synthetized Compositions	Initial V*^n^*^+^	M/P Ratio	V^2+^/V^3+^		V^3+^/V^4+^		V^4+^/V^5+^		Ref.
			E (V vs. Li^+^/Li)	Capacity (mAh/g)	E (V vs. Li^+^/Li)	Capacity (mAh/g)	E (V vs. Li^+^/Li)	Capacity (mAh/g)	
**Type I Materials**									
Na_3_V_2_(PO_4_)_3_	V^3+^	0.67	1.9 *	59	3.7 *	118	/	/	[39]
Na_3_V_1.5_Al_0.5_(PO_4_)_3_	V^3+^	0.67	1.9 *	60	3.7 *	85	4.3 *	28	[39]
Na_3_VCr(PO_4_)_3_	V^3+^	0.67	/	/	3.7 *	60	4.4 *	50	[42]
Na_4_VMn(PO_4_)_3_	V^3+^	0.67	/	/	3.7 *	60	4.2 *	50	[47]
r-Li_3_V_2_(PO_4_)_3_	V^3+^	0.67	/	/	3.7	131	/	/	[34]
m-Li_3_V_2_(PO_4_)_3_	V^3+^	0.67	1.8	131	3.9	131	4.5	33	[27]
LiVP_2_O_7_	V^3+^	0.5	2	116	4.3	95	/	/	[82,83]
Na_7_V_4_(P_2_O_7_)_3_(PO_4_)_2_	V^3+^	0.5	/	/	4.2 *	90	/	/	[84]
Na_7_V_3_Al_1_(P_2_O_7_)_3_(PO_4_)_2_	V^3+^	0.5	/	/	4.2 *	77	4.5	46	[85]
Na_3_V(PO_4_)_2_	V^3+^	0.5	/	/	3.8 *	90	4.4 *	20	[86,87]
LiV(HPO_3_)_2_	V^3+^	0.5	/	/	4.1	75	/	/	[88]
Li_9_V_3_(P_2_O_7_)_3_(PO_4_)_2_	V^3+^	0.375	/	/	3.7	55	4.5	55	[89]
Na_7_V_3_(P_2_O_7_)_4_	V^3+^	0.375	/	/	4.3 *	80	/	/	[90]
Na_3_V(PO_3_)_3_N	V^3+^	0.33	/	/	4.3 *	74	/	/	[91]
**Type II Materials**									
LiVPO_4_F	V^3+^	1	1.8	156	4.2	156	/	/	[14]
NaVPO_4_F	V^3+^	1	/	/	≈4.2 *	20	/	/	[92]
LiVPO_4_OH	V^3+^	1	1.4	155	/	/	/	/	[65]
(Li,K)VPO_4_F	V^3+^	1	/	/	4.0	110	/	/	[93]
Na_3_V_2_(PO_4_)_2_F_3_	V^3+^	0.67	1.5 *	64	4.0 *	64	≈ 4.8 *	64	[51,53,94]
Li_5_V(PO_4_)_2_F_2_	V^3+^	0.5	/	/	4.15	85	4.7	85	[95]
t-Na_5_V(PO_4_)_2_F_2_	V^3+^	0.5	/	/	3.7 *	62	/	/	[96]
o-Na_5_V(PO_4_)_2_F_2_	V^3+^	0.5	/	/	3.9 *	65	/	/	[96]
**Type III Materials**									
α-LiVPO_4_O	V^4+^	1	/	/	2.4	155	3.95	150	[14]
β-LiVPO_4_O	V^4+^	1	/	/	2.2	155	4	130	[97]
β-NaVPO_4_O	V^4+^	1	/	/	/	/	3.6 *	58	[98]
γ-LiVPO_4_O	V^4+^	1	/	/	2	80	4	150	[99]
Li_4_VO(PO_4_)_2_	V^4+^	0.5	/	/	2	94	4.1	94	[100]
Na_4_VO(PO_4_)_2_	V^4+^	0.5	/	/	/	/	3.8 *	77	[101]
Li_2_VOP_2_O_7_	V^4+^	0.5	/	/	/	/	4.1	64	[102]

The voltage values are reported vs. Li^+^/Li even for those obtained in Na-cell (according to E(Na^+^/Na) = 0.3 V vs. Li^+^/Li), in that case the voltage is marked by *

## Data Availability

Not applicable.
